# IQMNMR: Open source software using time-domain NMR data for automated identification and quantification of metabolites in batches

**DOI:** 10.1186/1471-2105-12-337

**Published:** 2011-08-12

**Authors:** Xu Song, Bo-Li Zhang, Hong-Min Liu, Bo-Yang Yu, Xiu-Mei Gao, Li-Yuan Kang

**Affiliations:** 1 Department of Chinese Medicinal Prescription, China Pharmaceutical University, Nanjing, China; 2 School of Pharmaceutical Sciences, Zhengzhou University, Zhengzhou, China; 3 State Key Laboratory of Modern Chinese Medicine, Tianjin University of Traditional Chinese Medicine, Tianjin, China

## Abstract

**Background:**

One of the most promising aspects of metabolomics is metabolic modeling and simulation. Central to such applications is automated high-throughput identification and quantification of metabolites. NMR spectroscopy is a reproducible, nondestructive, and nonselective method that has served as the foundation of metabolomics studies. However, the automated high-throughput identification and quantification of metabolites in NMR spectroscopy is limited by severe spectral overlap. Although numerous software programs have been developed for resolving overlapping resonances, as well as for identifying and quantifying metabolites, most of these programs are frequency-domain methods, considerably influenced by phase shifts and baseline distortions, and effective only in small-scale studies. Almost all these programs require multiple spectra for each application, and do not automatically identify and quantify metabolites in batches.

**Results:**

We created IQMNMR, an R package that integrates a relaxation algorithm, digital filter, and similarity search algorithm. It differs from existing software in that it is a time-domain method; it uses not only frequency to resolve overlapping resonances but also relaxation time constants; it requires only one NMR spectrum per application; is uninfluenced by phase shifts and baseline distortions; and most important, yields a batch of quantified metabolites.

**Conclusions:**

IQMNMR provides a solution that can automatically identify and quantify metabolites by one-dimensional proton NMR spectroscopy. Its time-domain nature, stability against phase shifts and baseline distortions, requirement for only one NMR spectrum, and capability to output a batch of quantified metabolites are of considerable significance to metabolic modeling and simulation.

IQMNMR is available at http://cran.r-project.org/web/packages/IQMNMR/.

## Background

Metabolomics, which complements other "omic" technologies (genomics, transcriptomics, and proteomics), is a rapidly emerging field of post-genomic research. One of the promising aspects of this discipline is metabolic modeling and simulation based on automated high-throughput identification and quantification of metabolites [1,2]. However, metabolomics does not feature well-defined methods for automated high-throughput identification and quantification of metabolites [3]. Until recently, numerous works on metabolomics have been restricted to qualitative studies, often the result of statistical model analysis rather than metabolic modeling and simulation [3,4].

NMR spectroscopy has served as the foundation of metabolomics studies [3]. The primary advantages of NMR spectroscopy are high reproducibility, non-destructiveness, non-selectivity in metabolite detection, and the ability to simultaneously quantify multiple classes of metabolites [5]. However, the automated high-throughput identification and quantification of metabolites in NMR spectroscopy is limited by severe spectral overlap [5].

Motivated by the requirement described above, researchers developed numerous software programs for automated resolution of overlapping signals, as well as metabolite identification and quantification; in these programs, one- or two-dimensional NMR spectra and databases of metabolite standards are used [6,7]. However, most of the existing software programs are frequency-domain methods, considerably affected by phase shifts and baseline distortions [3,5,6,8], and effective only in small-scale studies [7]. In addition, almost all these programs constantly require multiple spectra for each application, and do not automatically identify and quantify metabolites in batches [3,5,7].

In the current study, we created IQMNMR, an R package that provides one solution that can automatically identify and quantify metabolites by one-dimensional proton NMR spectroscopy. It differs from existing software in terms of the following aspects: it is a time-domain method, uninfluenced by phase shifts and baseline distortions; it uses not only frequency to resolve overlapping resonances but also relaxation time constants; and it requires only one NMR spectrum per application, but outputs a batch of quantified metabolites. These advantages are of considerable significance to metabolic modeling and simulation.

## Implementation

### Overview of program flow and critical issues

**Figure 1:**
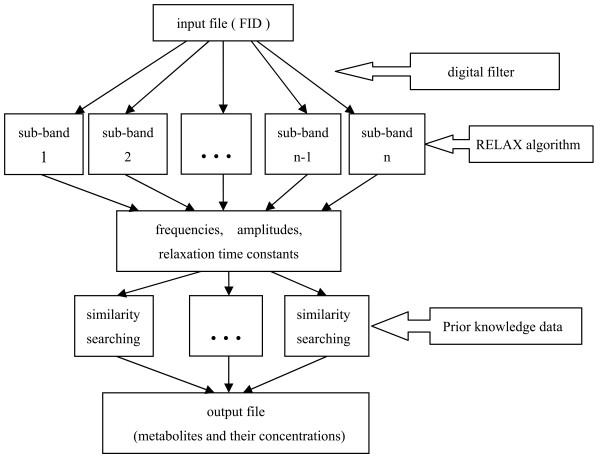
**The program flowchart of IQMNMR**. This is the program flowchart of IQMNMR. Relaxation algorithm and similarity search algorithm are parallelized.

IQMNMR is the integration of the RELAX algorithm (relaxation algorithm) [9], digital filter, and similarity search algorithm. Its program flowchart is presented in Figure 1.

IQMNMR uses the RELAX algorithm, which was first proposed by Li and Stoica in 1996 [9], to resolve overlapping signals. The algorithm assumes that the FID can be decomposed into *K *damped complex sinusoids.(1.1)

where *α_k_*, *d_k_*, and *ω_k _*represent the non-zero complex amplitudes, damping factors (inverse time constants), and frequencies; *z_k _*represents the signal poles; and *ξ*(*n*) denotes the unobservable additive noise.

Let(1.2)

The frequency and damping factor of the dominant peak of the FID can be computed by searching the maximum of . Then, complex amplitude *α_k _*can be calculated using .

With the above-mentioned procedures, the RELAX algorithm can be summarized as follows [10]:

Step 1. Assume that *K *= 1. Then, , , and  are obtained from *y*.

Step 2. Assume that *K *= 2. *y*_2 _is calculated with Eq. (1.2) using , , and  derived in Step 1. , , and  are then obtained from *y*_2_. Then, *y*_1 _is computed with Eq. (1.2) using , , and . We then re-determine , , and  from *y*_1_.

The first two steps are iterated until practical convergence is achieved (refer to the help files of IQMNMR).

Step 3. Assume that *K *= 3. *y*_3 _is computed with Eq. (1.2) using , , , , , and  obtained in Step 2. Subsequently, , , and  are derived from *y*_3_. Next, *y*_1 _is re-calculated with Eq. (1.2) using , , , , , and . , , and  are then re-determined from *y*_1_. After which *y*_2 _is re-calculated with Eq. (1.2) using , , , , , and , , , and  are re-determined from *y*_2_.

The previous steps are iterated until practical convergence is achieved (refer to the help files of IQMNMR).

The procedures are repeated until *K *is equal to the desired value (see the help files of IQMNMR).

Simulation examples and practical applications have demonstrated that the RELAX algorithm is accurate and robust [10,11]. The algorithm uses not only frequency to resolve overlapping resonances but also relaxation time constants [10], and has a resolution superior to that of FFT when FIDs are strongly damped or truncated [12]. As an iterative algorithm, however, its computational burden increases exponentially with the number of signals.

With the development of computer processor technologies, digital filtering has been increasingly used for NMR raw data processing [13]. A digital filter can suppress undesirable frequency ranges and maintain desired frequency ranges, as well as improve signal-to-noise ratio and overall sensitivity [13].

**Figure 2:**
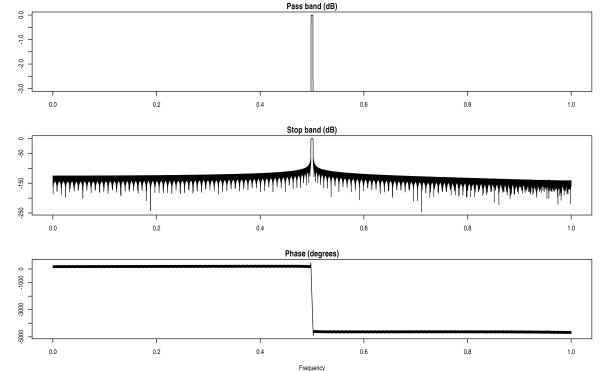
**The amplitude response and phase response of the digital filter**. This figure shows the amplitude response and phase response of the digital filter.

To reduce the heavy computational burden of the RELAX algorithm, a digital filter was integrated into IQMNMR. The digital filter is a symmetrical finite impulse response (FIR) bandpass filter. Figure 2 shows the amplitude response and phase response. The frequencies in the bandwidth of FID are modulated to the range of the passband before filtering, and then modulated back. Consequently, the input file (FID) is filtered into sub-bands.

As the input file (FID) is filtered into sub-bands, the total number of steps required by the RELAX algorithm decreases, and the computation could be parallelized. Parallel computing can be efficiently performed by cloud computing. An example is Amazon's Elastic Compute Cloud http://aws.amazon.com/ec2/, which was used in the field of comparative genomics[14]. In cloud computing, the time consumed by IQMNMR is substantially reduced. Digital filtering and cloud computing enable IQMNMR to be a high-throughput method.

After resolving each sub-band into damped sinusoids IQMNMR only keeps damped sinusoids that are within a specific frequency range. This range is less than the passband range of the sub-band to decrease the influence of the Gibbs effect, which stems from the digital filter. The passband range of each sub-band overlaps with that of adjoining sub-bands to avoid information loss.

Several metabolomic databases have emerged to serve as bioinformatics resources for identifying common metabolites from experimental data [15,16]. The Madison Metabolomics Consortium Database [16]http://mmcd.nmrfam.wisc.edu/, for instance, has collected information on more than 20,000 metabolites. Therefore, prior knowledge data sets containing the standard spectra of targeted metabolites can be created on the basis of these metabolomic databases.

The results of the RELAX algorithm are amplitudes, frequencies, and damping constants (the reciprocal of relaxation time constants). The initial time-domain amplitude of an NMR resonance is proportional to the frequency-domain area under the NMR spectral absorption mode peak. A cosine similarity measure [17] can be constructed on the basis of amplitudes (which are located in specific frequency ranges) and prior knowledge data sets. This way, the targeted metabolites are identified by the similarity search algorithm. The total number of hydrogen nuclei that generate the resonance lines of a targeted metabolite is directly proportional to the sum of integrated signal areas of the targeted metabolite. The targeted metabolites and internal standard are the components of the same sample, so that both have the same variation in receiver gain, probe design, etc. In this manner, the targeted metabolites can be quantified by comparing the amplitudes of the targeted metabolites and the internal standard.

### Workflow overview

IQMNMR is a fully automated method. Identifying and quantifying targeted metabolites entails only two steps.

### Step one: creating prior knowledge data sets of targeted metabolites

The prior knowledge data set consists of two tables: "lists_metabolites" and "space_x." The "lists_metabolites" table contains information on the molecular constitutions of targeted metabolites and experimental conditions of standard one-dimensional proton NMR spectroscopy. The "space_x" table contains information on the chemical shifts of targeted metabolites and area ratios of intra-molecular peaks. The variable descriptions of these tables are listed in the help files of IQMNMR.

We created a prior knowledge data set using the Madison Metabolomics Consortium Database as basis [16]. The aforementioned tables can be loaded by typing "data(lists_metabolites); data(space_x)" in the R command console. Furthermore, users can collect data and create prior knowledge data sets according to this paradigm.

### Step two: identifying and quantifying metabolites

The function "identify_quantify" uses the RELAX algorithm, digital filter, and similarity search algorithm to automatically resolve overlapping signals, as well as identify and quantify targeted metabolites. Its arguments are listed in the help files of IQMNMR. This function outputs a table that presents the names, concentrations, and cosine similarity measures of targeted metabolites.

## Results and Discussion

**Figure 3:**
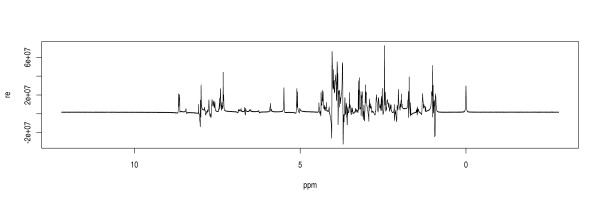
**The spectrum of simulated NMR experiment**. This is the spectrum of simulated NMR experiment. The magnetic field strength was set to 400 MHz. The internal standard is DSS (4,4-dimethyl-4-silapentane-1-sulfonic acid). The solvent is water.

A simulated one-dimensional proton NMR experiment was carried out to illustrate the functionality of IQMNMR. IQMNMR provides four functions: "select_metabolites," "NMR_experiment," "NMR_spectra," and "identify_quantify" for users to select metabolites and true concentrations, generate simulated FID, present NMR spectrum, and identify and quantify targeted metabolites. Figure 3 shows the simulated NMR frequency spectrum. Table 1 shows the true concentrations, measured concentrations, and related errors. The relative error is defined as follows:(1.3)

**Table 1:** The results of identification and quantification

Name	Measured Concentration (mM)	True Concentration (mM)	Relative error
Acetic acid	0	1.91	

Adonitol	0	0	

Agmatine	23.94	27.76	13.76

Alanine	0	0	

*beta*-Alanine	8.34	14.08	40.77

*alpha*-Ketoglutaric acid	1.83	1.81	1.27

Methyl 4-aminobutyrate	8.70	10.95	20.50

4-(2-Aminoethyl)morpholine	0	0	

Anthranilic acid	0	0	

L-Arginine	0	0	

L-Ascorbate	0	0	

L-Asparagine	17.39	21.83	20.34

Benzoate	0	0	

*trans*-Cinnamic acid	7.22	5.086	42.03

Citrate	3.57	2.92	22.15

Ethanol	0	0	

D-Galactono-1,4-lactone	0	24.73	

L-Glutamic acid	0	0	

L-Histidine	0	0	

Homogentisic acid	0	0	

*O*-Succinyl-L-homoserine	0	0	

Imidazole	0	0	

Inosine 5'-monophosphate	0	0	

L-Isoleucine	20.35	21.03	3.25

L-Kynurenine	8.048	5.48	46.76

Malic acid	22.10	27.65	20.10

*N*-Acetyl-D-mannosamine	10.56	17.90	41.02

L-Methionine methylsulfonium iodide	9.60	8.84	8.64

3-Methyl-2-oxobutanoic acid	0	0	

Nicotinic acid	0	0	

Nicotine	12.20	8.10	35.75

4-Nitrocatechol	15.70	12.02	30.65

*N(alpha)*-Acetyl-DL-ornithine	0	0	

Phenol	0	0	

Phenylacetic acid	0	0	

L-Phenylalanine	10.85	21.75	50.10

DL-Pipecolic acid	0	0	

Polygalacturonic acid	0	0	

L-Proline	0	0	

*trans*-4-Hydroxy-L-proline	0	0	

Pyridoxal-5-phosphate	41.34	19.61	110.78

Quinolinic acid	16.69	16.51	1.04

D-Ribulose 5-phosphate	0	0	

Sarcosine	0	0	

L-Serine	0	0	

L-Threonine	10.45	13.12	20.37

D-Trehalose	0	0	

Trigonelline	0	0	

Tryptamine	0	0	

Tyramine	0	0	

L-Tyrosine	0	0	

Uracil	0	0	

Uridine	8.28	10.97	24.53

L-Valine	18.66	13.66	36.60

where "*m*" and "*r*" are the measured and real concentrations of targeted metabolites, respectively. The identification rate is defined as the number of identified metabolites divided by the total number of targeted metabolites. A metabolite is identified if its true and measured concentrations are higher than zero, or if its true and measured concentrations equal zero.

Figure 3 shows clear phase shifts and baseline distortions. As a time-domain method, IQMNMR is stable against phase shifts and baseline distortions. Table 1 presents the result of IQMNMR. The mean of related errors is 29.52%; the standard deviation of related errors is 23.70%; and the identification rate is 96.36%. Given that FID is filtered into sub-bands and the computation is parallelized, cloud computing [14] can substantially reduce the time consumed by IQMNMR. On the basis of these results, we conclude that IQMNMR provides one solution that can automatically identify and quantify metabolites in batches.

Quantification in metabolomics is generally performed by either absolute or relative quantification. Absolute quantification uses an internal standard to determine the absolute concentration. The metabolites and internal standard are the components of the same sample. Hence, changes in receiver gain, probe design, etc. are the same for the metabolites and internal standard. The signal intensities in an NMR spectrum only depend on the molar concentrations of the sample [18]. Consequently, the absolute concentrations of metabolites can be easily obtained after using RELAX and similarity search algorithms by comparing the amplitudes of the targeted metabolites and the internal standard. In relative quantification, the metabolite signal intensity is normalized to that of a specific metabolite, which is the component of the same sample. In principle, absolute quantification encompasses relative quantification. If the absolute concentrations of the metabolites are known, their relative ratios can be easily calculated. Additionally, for relative quantification, an accurate determination of the internal standard concentration is unnecessary.

The quantitative error is affected by color noise, white noise, the Gibbs effect of a digital filter, and signal overlapping. The RELAX algorithm performs well in the presence of colored noise, white noise, and signal overlapping [10,11]. However, this algorithm is unable to deal with the quantitative error caused by the Gibbs effect. Oversampling technique had been used in modern NMR spectrometry [13,19,20]. Oversampling can ensure a higher filter order, and consequently decrease the ripple and proportion of the overshoot range in the passband range. Therefore, oversampling can effectively deal with the influence of the Gibbs effect. However, the final FID generated by modern NMR spectrometry is reduced in order to avoid a large data set. For example, in 20-fold oversampling, the number of data points also increases by a factor of 20 [13]. For an FID size of 64 000 data points, 20-fold oversampling results in 1.3 million data points [13].

Presently, IQMNMR only uses information on amplitude ratios and peak locations. In future editions, information on coupling will be used. We believe that coupling information decreases identification and quantification errors.

To highlight the resolution of the RELAX algorithm, the magnetic field strengths of the simulated FID cited above were set to 400 MHz. Some metabolomics studies were carried out at low magnetic field strengths (<600 MHz) [21], but a higher magnetic field leads to increased signals resolution, thereby improving the performance of the RELAX algorithm. We suggest that higher magnetic fields be used to generate FIDs for the application of IQMNMR.

Different NMR spectrometers must use different prior knowledge data sets acquired at the same magnetic field strengths as the NMR spectrometer settings. Before using this package, users must create a prior knowledge data set that matches the magnetic field strength of their NMR spectrometer.

Some unknown metabolites will inevitably exist in the sample. IQMNMR assumes that FID is modeled as the sum of sinusoidal, autoregressive noise, and white gaussian noise signals. Whether these signals are known, the digital filter separates FID into sub-bands, the RELAX algorithm decomposes these sub-bands into their constituent signals, and the similarity search algorithm identifies the signal combinations that match the prior knowledge data set and quantifies them. Future editions will involve the generation of resultant NMR data that contain only the remaining sinusoidal and noise signals, so that further analysis can be performed by users.

IQMNMR reduces spectral data to a batch of quantified metabolites that is more beneficial than spectral binning. The batch of metabolites can be directly used as input variables in principal component analysis or metabolic modeling and simulation.

Although IQMNMR provides for metabolomics identification and quantification, validation via application to real samples (i.e., complex multicomponent systems) should be a prerequisite for practicality. Metabolomics reflects a paradigm shift from reductionism to holism [22]. The key to its success is multi-disciplinary collaboration [22].

## Conclusions

Metabolite identification is the foundation of metabolomics. The quantification of metabolites is a state-of-the-art approach. IQMNMR provides one solution that can automatically identify and quantify metabolites in batches by one-dimensional proton NMR spectroscopy. It is a time-domain method that features stability against phase shifts and baseline distortions. It uses not only frequency to resolve overlapping resonances but also relaxation time constants. It requires only one NMR spectrum per application, and produces a batch of quantified metabolites. These features are of considerable significance to metabolic modeling and simulation.

## Competing interests

The authors declare that they have no competing interests.

## Availability and requirements

Project name: IQMNMR

Project home page: http://cran.r-project.org/web/packages/IQMNMR/

Operating systems: UNIX or MAC

Programming language: R

Other requirements: None

License: GNU GPL

Any restrictions on use by non-academics: None

## List of abbreviations

NMR: nuclear magnetic resonance; FID: free induction decay; RELAX algorithm: relaxation algorithm; FFT: fast Fourier transform

## Authors' contributions

BZ, HL and XS conceived the strategies. BZ and HL supervised this project. XS, BY, XG, and LK discussed and designed this software. All authors contributed equally to this work. All authors read and approved the final manuscript.
